# Children's Acquisition of the English Past‐Tense: Evidence for a Single‐Route Account From Novel Verb Production Data

**DOI:** 10.1111/cogs.12581

**Published:** 2018-01-12

**Authors:** Ryan P. Blything, Ben Ambridge, Elena V.M. Lieven

**Affiliations:** ^1^ School of Psychological Science University of Bristol; ^2^ University of Liverpool ESRC International Centre for Language and Communicative Development (LuCiD); ^3^ University of Manchester ESRC International Centre for Language and Communicative Development (LuCiD)

**Keywords:** Cognitive development, Inflectional morphology, Past‐tense, First language acquisition, Analogy

## Abstract

This study adjudicates between two opposing accounts of morphological productivity, using English past‐tense as its test case. The single‐route model (e.g., Bybee & Moder, 1983) posits that both regular and irregular past‐tense forms are generated by analogy across stored exemplars in associative memory. In contrast, the dual‐route model (e.g., Prasada & Pinker, 1993) posits that regular inflection requires use of a formal “add ‐*ed*” rule that does not require analogy across regular past‐tense forms. Children (aged 3–4; 5–6; 6–7; 9–10) saw animations of an animal performing a novel action described with a novel verb (e.g., *gezz; chake*). Past‐tense forms of novel verbs were elicited by prompting the child to describe what the animal “did yesterday.” Collapsing across age group (since no interaction was observed), the likelihood of a verb being produced in regular past‐tense form (e.g., *gezzed; chaked*) was positively associated with the verb's similarity to existing regular verbs, consistent with the single‐route model only. Results indicate that children's acquisition of the English past‐tense is best explained by a single‐route analogical mechanism that does not incorporate a role for formal rules.

## 1 Introduction

A major debate among cognitive scientists is whether a speaker's ability to produce novel sentences and morphologically inflected forms should be attributed to symbolic rules that act over abstract categories (e.g., Chomsky, 1965; Prasada & Pinker, 1993) or to a mechanism that analogizes across stored exemplars (e.g., Bybee & Slobin, 1982; Rumelhart & McClelland, 1986). English past‐tense morphology, which is characterized by its clear distinction between regular (e.g., *kick/kicked; play/played*) and irregular (e.g., *keep/kept; steal/stole*) patterns of inflection, provides a particularly suitable framework to adjudicate between these two approaches. Specifically, the emergence of “overregularization” errors in children's speech (when regular inflections are applied to verbs that require irregular inflection; e.g., *keeped, *stealed, *hitted) has given rise to two opposing accounts of children's morphological productivity: the *dual‐route* model (e.g., Prasada & Pinker, 1993), which posits that overregularization reflects the use of a formal morphological rule (i.e., add “–*ed*”), and the *single‐route* model (e.g., Bybee & Moder, 1983), which posits that overregularization reflects analogy across stored exemplars (e.g., *peel/peeled, heal/healed*, steal/*stealed). This study uses an elicited production paradigm to investigate which of these assumptions best accounts for young children's morphological productivity.

For irregular past‐tense forms (e.g., *kept, stole*), the single‐ and dual‐route models assume, in effect, identical mechanisms: Both accounts assume that these forms are stored in associative memory and used in analogical generalization. If an irregular form cannot be retrieved directly from memory (i.e., if the representation is weak or absent), it is generated by analogy to phonologically similar stored forms (e.g., if the past‐tense of *know* has not yet been learned, *knew* may be generated by analogy to *blow/blew* and *throw/threw*). Thus, with regard to novel‐verb studies, the single‐ and dual‐route models make an identical prediction: The likelihood of a novel verb being produced in irregular past‐tense form is positively associated with its phonological similarity to existing irregular verbs. Indeed, this prediction is supported in elicitation studies with adults (e.g., Albright & Hayes, 2003; Prasada & Pinker, 1993), as well as in a judgment task with children (Ambridge, 2010). However, since the single‐ and dual‐route models make identical predictions with regard to the effect of similarity‐to‐irregulars, it does not help to adjudicate between the two accounts.

To do so, we need to investigate a phenomenon for which the two accounts make different predictions: production of regular inflection. The single‐route model (e.g., Bybee & Moder, 1983) holds that all morphological productivity—both regular and irregular—can be attributed to a single associative process (essentially the same process that the dual‐route model assumes for irregulars only). If a verb's (regular or irregular) past‐tense form cannot be retrieved directly from memory (e.g., *knew*), a past‐tense form is generated by analogy to phonologically similar verbs *regardless of their regularity* (e.g., *knew* may be generated by analogy with irregular verbs like *throw/threw* and *blow/blew* but *knowed may be generated by analogy with regular verbs like *show/showed* and *glow/glowed*). Thus, the single‐route model predicts that the likelihood of a novel verb being produced in regular past‐tense form is positively associated with its phonological similarity to existing regular verbs.

Under the dual‐route model (Prasada & Pinker, 1993) regular forms are generated via the application of a default rule that adds the suffix “–*ed*” to a verb's root‐form (e.g., *know*/*knowed).^1^ This default rule steps in whenever an irregular form fails to be either retrieved directly from memory or generated by analogy with stored irregulars. Crucially, the “add –*ed*” rule can be applied to any verb “regardless of anything else that might happen to the stem as a result of other rules or memorized associations” (Berent, Pinker, & Shimron, 2002, p. 463). Thus, the dual‐route model does not share the prediction of the single‐route model that the likelihood of a novel verb being produced in regular past‐tense form is positively associated with its phonological similarity to existing regular verbs. Rather, Prasada and Pinker (1993, p. 22) predict that “the goodness of the suffixed past‐tense forms does not decline as a function of distance from known suffixed forms.” Indeed, Prasada and Pinker (1993) take this very finding (from their adult judgment study) as evidence for the dual‐route model.

There have been attempts to modify the dual‐route model to allow for storage of at least some high‐frequency regulars (e.g., Alegre & Gordon, 1999; Pinker & Ullman, 2002). In our view, such modification renders the model empirically indistinguishable from the single‐route model. We come back to this in the Discussion, but for now, we focus on the clearly contrasting predictions of each account. In summary, setting aside irregulars (for which the models share an identical prediction) and modified versions of the dual‐route model (whose predictions are less straightforward), the contrasting prediction of the accounts is clear: The single‐route model predicts a positive association between the likelihood of a novel verb being produced in regular past‐tense form and its phonological similarity to existing regular forms. The dual‐route model does not. Note that it is generally agreed that this prediction must be tested with novel verbs (e.g., Albright & Hayes, 2003; Ambridge, 2010; Prasada & Pinker, 1993) which require the use of a generalization mechanism (i.e., either rules or analogy) rather than allowing for the retrieval of rote‐learned forms from memory (assumed under both accounts).

Two previous novel‐verb studies have challenged the claim of the dual‐route model that regular inflection requires a context‐free “add –*ed*” rule. Albright and Hayes (2003) concatenated “combinations of relatively common syllable onsets and syllable rhymes” (p. 135) in 4,253 English verbs to create novel verbs that varied in the extent to which they were phonologically similar/dissimilar to existing regular and irregular verbs. Consistent with predictions of both single‐route and dual‐route models, adult English speakers were more likely to produce and favorably judge irregular past‐tense forms of novel verbs that were similar to existing irregulars. Crucially, participants were also more likely to produce and favorably judge regular past‐tense forms of novel verbs that were similar to existing regulars, consistent with the predictions of the single‐route but not the dual‐route model.

Second, Ambridge (2010) replicated the judgment component of Albright and Hayes (2003) with children aged 6–7 and 9–10. For both ages, the acceptability of novel irregular past‐tense forms increased as a function of the verb's similarity to existing irregular verbs (consistent with both models). However, consistent with the predictions of the single‐route model only, acceptability of novel regular past‐tense forms increased as a function of the verb's similarity to existing regular verbs (though for the older group only).

The novel verb findings of Albright and Hayes (2003) and Ambridge (2010) constitute preliminary support for the single‐route over the dual‐route model. However, at present, this support must be considered tentative for two key reasons. First, evidence for the crucial effect of similarity‐to‐regulars has been observed only in children aged 9–10 (Ambridge, 2010) and adults (Albright & Hayes, 2003; Ambridge, 2010). The main problem here is that these populations are well past the peak rate of overgeneralization (e.g., Maratsos, 2000; Maslen, Theakston, Lieven, & Tomasello, 2004; Stemberger, 1993), meaning that the data reflect the output of relatively mature systems rather than tapping directly into acquisition. Thus, it is important to test younger children who, unlike the age groups outlined above, are still in the initial stages of learning to produce past‐tense forms. Younger children are also important to test because 9–10 year olds and adults may well be subject to demand characteristics (in both production and judgment studies). For example, they might assume that the experimenter does not intend for them to produce—or give maximum acceptability ratings to—regular forms on every trial, and so base their regular productions and judgments on analogy to known regular forms, even though they may not necessarily do so in normal circumstances.

The second reason why current evidence must be considered tentative is that those (9‐ to 10‐year‐old) children who have shown the crucial effect of similarity‐to‐regulars have done so only with judgment data. The problem here is that overregularization errors are a production phenomenon, and providing acceptability judgments can be argued to be a metalinguistic task. Any demonstration of the similarity‐to‐regulars effect would be more convincing if a more ecologically valid task such as production were used. Production tasks may also be more suitable for testing young children. For example, the 6‐ to 7‐year olds studied by Ambridge (2010) failed to show a significant effect of similarity‐to‐regulars (despite an effect of similarity‐to‐irregulars), and it is difficult to know whether this pattern reflects this age group's relatively incomplete formation of phonological neighborhoods for regular verbs (which generally have lower token frequency than irregular verbs), or difficulties with the judgment task. Note that such difficulties need not necessarily affect regular and irregular forms equally (e.g., perhaps some children simply gave higher ratings to regulars across the board). To date, production studies that have investigated children's acquisition of past‐tense have been limited to real verb stimuli. For example, Marchman (1997) (see also Marchman, Wulfeck, & Weismer, 1999) used an elicited production study to investigate children's (aged 3;8–13;5) zero‐marking errors with real English verbs (e.g., supplying *try* as the past‐tense of *try*). Collapsing across regular and irregular verbs, verbs which were phonologically similar to a high number of other verbs (e.g., *pick*) were more resistant to zero‐marking errors than verbs which were phonologically similar to few verbs (e.g., *melt*). Since there was no interaction of this effect with regularity of the verb, the finding was taken as evidence that children analogize to stored regular past‐tense forms. However, a confound of studies that employ real verbs as test stimuli is that use of these verbs is affected by factors like memory and frequency. For this reason, it is generally agreed that predictions of the single‐route and dual‐route models should be tested with novel verbs (e.g., Albright & Hayes, 2003; Prasada & Pinker, 1993), for which children must use their capacity to generalize (i.e., either rules or analogy) as opposed to relying on rote‐learned forms.

In sum, evidence for the crucial effect of similarity‐to‐regulars requires supporting data from (i) age groups who are in the initial stages of learning to produce past‐tense forms, (ii) production tasks, and (iii) novel verb stimuli. For these reasons, this study employed an elicited production paradigm using novel verbs (the core set from Albright & Hayes, 2003) to investigate the generalization mechanisms underlying 3–4, 5–6, 6–7, and 9–10 year olds' productivity with English past‐tense. Novel verbs were elicited using a sentence‐completion task (e.g., *The duck likes to bredge. Look, there he is bredging. Everyday he bredges. So yesterday he…*). The central aim of this study is to test the crucial contrasting predictions of the two accounts: The single‐route model predicts a positive association between the likelihood of a novel verb being produced in regular past‐tense form and its phonological similarity to existing regular forms; the dual‐route model predicts no such effect.

## 2 Method

### 2.1 Participants

Eighteen children were recruited from each age group (3–4 [M = 3;8], 5–6 [M = 5;9], 6–7 [M = 7;3], and 9–10 [M = 10;4] year olds). An additional three children from the youngest age group were excluded because they did not comply with procedure. All participants were monolingual English speakers and had not been diagnosed with any language impairment. All participants were recruited from schools or nurseries in Manchester and testing occurred at those locations in a private room.

### 2.2 Materials

The study used 40 novel verbs (e.g., *bize, rife, chool, spling*), all sourced from Albright and Hayes's (2003) study.^2^ To ensure a fair test of the predictions of the single‐route model and to ensure findings are compatible with alternatives present in the literature, the study employed two different measures of novel verbs' graded similarity to existing regular past‐tense verbs. Namely, the Generalized Context Model (GCM; Nosofsky, 1990) and the Minimal Generalization Learner (MGL; Albright & Hayes, 2003) provide measures of “variegated” similarity and “structured” similarity, respectively (each described below), both of which are compatible with predictions of the single‐route but not the dual‐route model.

#### 2.2.1 “Variegated” similarity measure (analysis 1)

The first measure of phonological similarity to existing verbs was obtained from Albright and Hayes's (2003) implementation of GCM (Nosofsky, 1990), which registered the phonological properties of 4,253 existing verbs' stem and past‐tense form (4,035 regular; 218 irregular) regardless of the verb's regularity. GCM outputs a well‐formedness score for a novel verb's past‐tense form using the following procedure. First, the phonological change required for the novel verb to take its past‐tense form is identified (e.g., the phonological change required for *scride* to become *scrode* is [aɪ]→[o]). Second, the model identifies all existing verbs that undergo the same phonological change (e.g., *stride→strode; rise→rose; write→wrote; dive→dove; ride→rode,* etc.) and calculates the phonological similarity of the novel verb to each of those verbs separately. Similarity is analyzed across the entire word by comparing each phonological segment of the novel and comparison verb, using an estimate of relative similarity proposed by Broe (1993) (see Albright & Hayes, 2003). Finally, the novel verb's similarity scores to each comparison verb are summed to provide an overall well‐formedness score. The model output well‐formedness scores for each novel verb's irregular and regular past‐tense forms, and these scores are used as a measure of the verb's similarity to existing irregular and regular verbs, respectively.

#### 2.2.2 “Structured” similarity measure (analysis 2)

The alternative measure of phonological similarity used in the analyses is one of “structured” similarity and is obtained from MGL (Albright & Hayes, 2003). MGL registers the phonological properties of every encountered verb stem and its past‐tense form (regardless of regularity) and forms “micro‐rules” that describe each (word‐specific) phonological change. Through a process known as *minimal generalization,* MGL generalizes over phonological contexts in which existing verbs undergo the same micro‐rule (for full description, see Albright & Hayes, 2003), and outputs graded confidence values for a verb's past‐tense forms based on the reliability of the context‐dependent micro‐rule which produces that form. For example, a micro‐rule that outputs regular past‐tense forms (e.g., ø→əd) based on a specific phonological context (e.g., “verb must end in [d] or [t]”) is more reliable if a high proportion of existing verbs that meet this context have the same regular form (‐əd) (e.g., *wan**t**, nee**d**, star**t**, deci**de**,* etc./*ge**t**, *stan**d**, etc.). The measure is termed “structured” similarity because of the requirement for all comparison verbs (i.e., verbs to be analogized to) to share not just the same phonological change (e.g., ø→əd) (as under variegated measures of similarity) but also a uniformed phonological structure (e.g., verbs stem must also end in [d] or [t]).

Note that “variegated” and “structured” measures of similarity are compatible with analogy‐based, single‐route models in different ways. Variegated similarity is considered by some as a “pure” form of analogy (e.g., Albright & Hayes, 2003, p. 122) because it works on the basis of comparing phonological segments across entire words such that phonological similarities at the beginning, middle, and end of a word have equal weighting, and each comparison form can be similar to the novel verb in its own way. Structured similarity is compatible with more sophisticated conceptions of analogy (such as schema‐based approaches; e.g., Langacker, 2000) in which analogy is permitted to focus on particular phonological properties. Crucially, both measures can be used to test whether regular past‐tense forms are produced by analogizing across stored exemplars of existing regular verbs. Since testing this prediction is the main interest of the present paper, we chose not to favor one similarity measure over another but instead use both measures to corroborate findings.

### 2.3 Design

Each participant was presented with 40 verbs in a within‐subjects design. To combat fatigue effects, verbs were equally divided into Set “A” and “B,” to be completed on different days (see *Appendix* 1 for classification). Children were assigned to one of two counterbalanced groups which ensured that half were first exposed to Verb‐Set A (followed by B on a different day) and the remaining half were first exposed to Verb‐Set B (followed by A on a different day).

The predictor variables were (a) phonological similarity to existing regular verbs, (b) phonological similarity to existing irregular verbs—both continuous predictors—and (c) participant age group (3–4, 5–6, 6–7, 9–10 years). The (trial‐level) outcome variable was whether the novel verb was produced in regular form (e.g., *bized*) or irregular form (e.g., *boze*). Since responses that did not constitute past‐tense forms were excluded from the denominator (see *Results*), the outcome variable provided a measure of the likelihood of both regular (vs. irregular) and irregular (vs. regular) production of past‐tense forms.

### 2.4 Procedure

Children were told that the experimenter would describe cartoons on the laptop and that they should “fill‐in‐the‐blanks” when the experimenter stopped talking. This format was designed to elicit plural forms of nouns in the practice session and past‐tense forms of verbs in the test session.

#### 2.4.1 Practice session

For each practice trial, the experimenter described the first image of a single item (e.g., “*Here is one mouse*”) and then began to describe a second image that depicted more than one instance of that item (“*and now there are three*…”). In order to ensure that the child was comfortable with producing both regular and irregular responses, two of the four practice trials used nouns with regular plural forms (*shoe/shoes; car/cars*) and the remaining two trials displayed images of nouns with “irregular” plural forms (*man/men; mouse/mice*). Overregularization errors (e.g., “*mouses*”) were corrected by the experimenter.

#### 2.4.2 Test session

The child was told that she would see cartoon videos of animals “doing some funny things.” Forty video animations were created using *Anime Studio Pro 6*, each featuring one of four animals (*duck, bunny, frog, or bear*) performing a novel intransitive action. Animations were played using *Apple QuickTime*. Each trial presented a verb in bare‐stem (non‐finite), present progressive (‐*ing*), and simple present‐tense form (‐s), using the following template: “*The duck/bear/frog/bunny likes to VERB. Look, there he is VERBing. Everyday he VERBs. So yesterday he…*” Animation‐verb pairings were random, and different for each participant, in order to control for any possible semantic effects on the use of regular versus irregular past‐tense forms (e.g., Ramscar, 2002). Verbs were presented in pseudo‐random order, different for each participant, with the constraint that no more than two consecutive trials featured a verb from the same “island of reliability” (using Albright and Hayes's classification of verbs similar to existing regular and/or irregular verbs).

## 3 Results

A total of 2,880 trials were recorded (720 for each age group). Responses were coded according to whether the response was a (i) Regular Form (e.g., *bized*), (ii) Irregular Form^3^ (e.g., *boze*), (iii) No‐change (e.g., *bize*), (iv) Third‐person‐present (e.g., *bizes*), (v) Past progressive (e.g., *bizing*), or (vi) Unclassified (i.e., a response that could not be classified as (i) to (v)). The mean proportion of each response, by age group, is shown in Table 1. Given the study's focus on the past‐tense system, only regular and irregular forms were analyzed, and all other responses were excluded. Thus, the statistic in Table 1 that corresponds to the (trial‐level) outcome measure is *mean percentage of regular vs. irregular responses only* (shown in bold font), which indicates the likelihood of regular (vs. irregular) and irregular (vs. regular) production of past‐tense forms.

**Table 1:** Mean percentage (SD in brackets) of regular, irregular, no change, third‐person present, past progressive, and uncategorized forms produced by each age group

	Regular (vs. Irregular Only**)**	Regular (vs. all)	Irregular (vs. all)	No Change (vs. all)	Third‐Person Present (vs. all)	Past Progressive (vs. all)	Uncategorized (vs. all)
3–4 years	**0.88**	0.27	0.04	0.07	0.36	0.07	0.19
	**(0.33)**	(0.44)	(0.19)	(0.26)	(0.48)	(0.26)	(0.39)
5–6 years	**0.95**	0.38	0.02	0.02	0.47	0.02	0.10
	**(0.22)**	(0.48)	(0.14)	(0.15)	(0.50)	(0.13)	(0.30)
6–7 years	**0.96**	0.83	0.04	0.01	0.02	0.06	0.04
	**(0.20)**	(0.38)	(0.19)	(0.10)	(0.14)	(0.24)	(0.20)
9–10 years	**0.94**	0.81	0.06	0.01	0.01	0.03	0.08
	**(0.24)**	(0.39)	(0.23)	(0.11)	(0.07)	(0.16)	(0.28)
All ages	**0.94**	0.57	0.04	0.03	0.21	0.04	0.10
	**(0.24)**	(0.49)	(0.19)	(0.17)	(0.41)	(0.20)	(0.31)

### 3.1 Analysis 1

**Figure 1:**
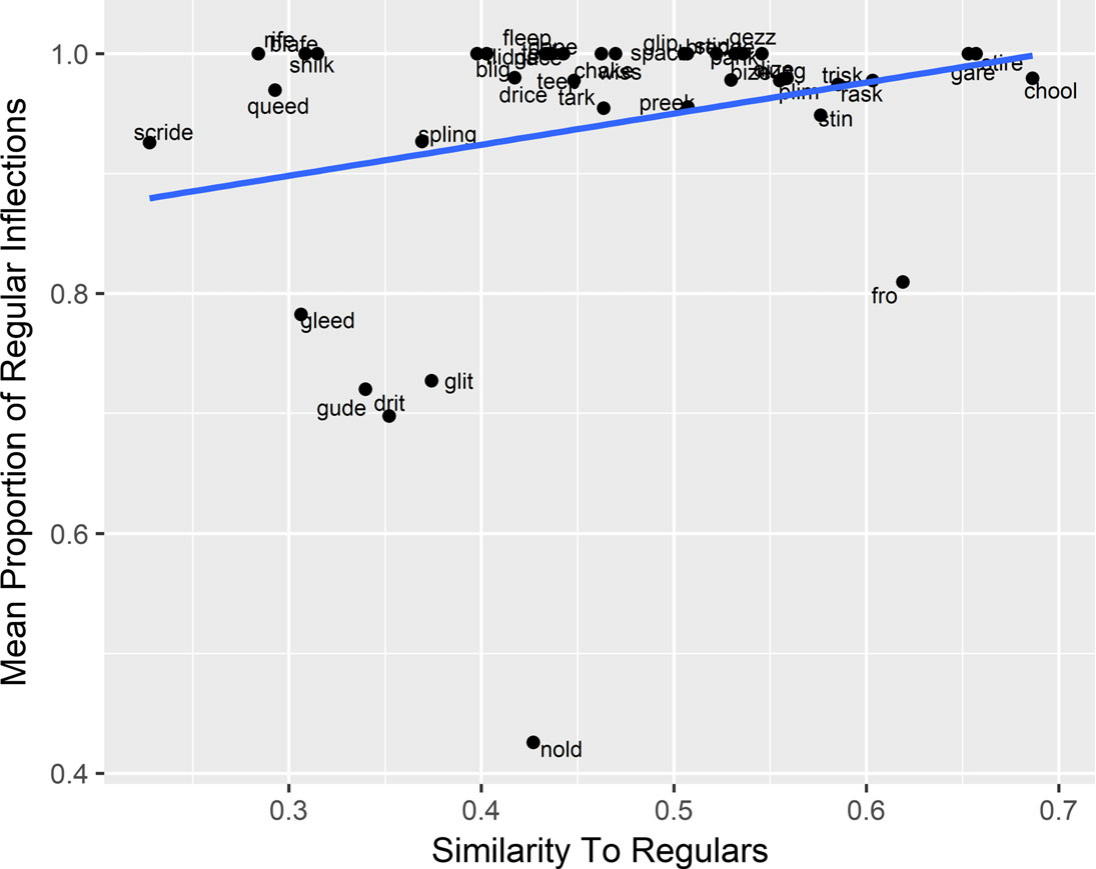
Mean proportion of verbs that received regular inflection as a function of their similarity to existing regular verbs (collapsing across age).

**Figure 2:**
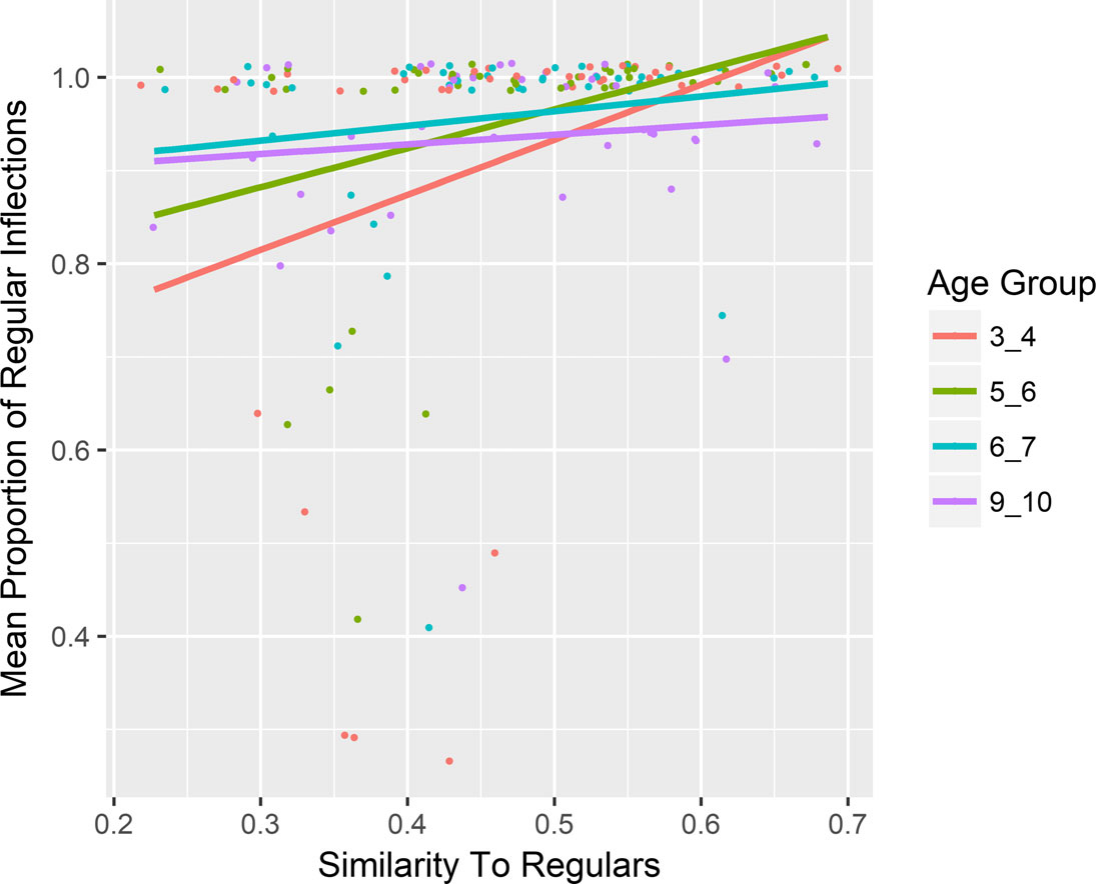
Mean proportion of verbs that received regular inflection as a function of their similarity to existing regulars (by age group).

Fig.** 1 plots the rate of regular and irregular inflection as a function of similarity to existing regulars (collapsing across age). Fig.** 2 plots this relationship for each age group separately (data‐points for each age group are color‐coded). Both figures suggest that, as predicted by the single‐route model only, the likelihood of a verb being produced in regular past‐tense form is positively associated with its phonological similarity to existing regular verbs.

**Figure 3:**
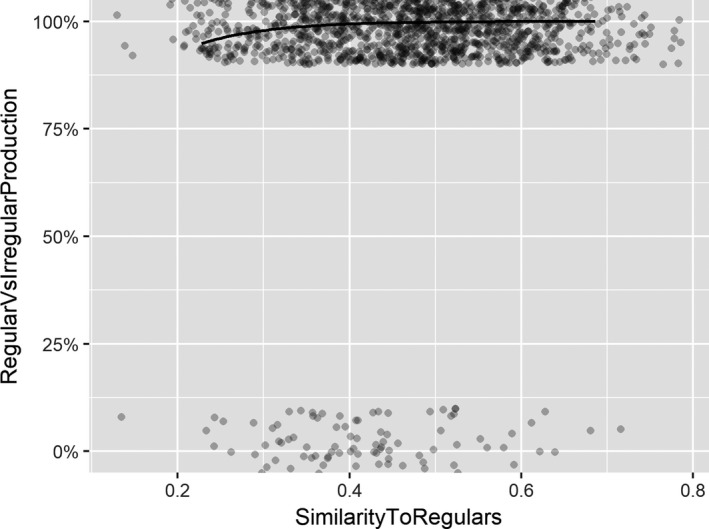
Plot of glmer model to show predicted rate of regular inflections as a function of similarity‐to‐regulars fixed effect.

To investigate whether this trend was statistically significant, results were analyzed using binomial linear mixed‐effects models (*glmer* from package *lme4;* Bates, Maechler, Bolker, & Walker, 2015) in the R environment (R Development Core Team, 2011). The main advantages of mixed‐effects models are that they predict individual trials rather than average over trials,^4^ are robust against missing data, and treat both participants and items as random effects (Baayen, Davidson, & Bates, 2008). The analysis used as its outcome variable *production of regular forms (vs. irregular forms)*. Fixed effects were entered into the model simultaneously (thus, the order in which predictors are listed is arbitrary): (a) *age* (centered), (b) *similarity to existing irregulars,* (c) *similarity to existing regulars*, (d) age*similarity to existing irregulars, and (e) age*similarity to existing irregulars. The model included random intercepts for participants and verbs, and random slopes where applicable (following recommendations outlined in Barr, Levy, Scheepers, & Tily, 2013). Since the model did not converge when fitted with random slopes that corresponded to more than one fixed effect, the model was simplified to include by‐participant random slopes only for the critical variable of interest: similarity‐to‐regulars (Barr et al., 2013). Random‐intercepts offer the benefit of removing idiosyncratic variation within each random factor (i.e., participants and verbs), whereas random‐slopes control for the possibility that treatment effects may vary within each random factor. Fig*.* 3 visualizes the fitted *glmer* model, using the *spj.glmer* function from the *sjPlot* package (Lüdecke, 2017) to plot the probability curve for the similarity‐to‐regulars fixed effect.

The output of the model is shown in Table 2. The model did not show a significant effect of *similarity‐to‐irregulars* (although the effect was in the predicted direction), nor did this effect interact with *Age*. Crucially, and in support of the single‐route model only, a main effect of *similarity‐to‐regulars* was observed: The likelihood of a verb's being produced in regular past‐tense form was positively associated with its phonological similarity to existing regular verbs. The effect of *similarity‐to‐regulars* significantly interacted with *Age,* and the negative *B* value for this effect (Table 2) indicates that the effect decreased with age (i.e., the effect was more pronounced in younger children).^5^

**Table 2:** Mixed‐effects models fitted to production of regular forms (“variegated” similarity measures obtained from GCM)

	Beta (B)	*SE*	z	2.5%	97.5%	Pr(>|z|)
(Intercept)	0.90	2.40	0.38	−3.80	5.60	0.707
Age	1.11	0.36	3.05	0.40	1.82	0.002
Similarity‐to‐irregulars	−11.58	12.69	−0.91	−36.45	13.30	0.362
Similarity‐to‐regulars	**11.99**	**5.13**	**2.34**	**1.94**	**22.05**	**0.019**
Age*similarity‐to‐irregulars	−1.76	1.69	−1.04	−5.08	1.55	0.298
Age*similarity‐to‐regulars	−**2.25**	**0.88**	−**2.56**	−**3.98**	−**0.52**	**0.011**

Boldface type indicates significant effect.

### 3.2 Analysis 2: Structured versus variegated similarity in analogy

At this point, it is important to consider possible shortcomings of the “variegated” similarity metric used in *Analysis 1*, which was obtained from GCM (Nosofsky, 1990). GCM works on the basis of comparing phonological segments across entire words such that phonological similarities at the beginning, middle, and end of a word have equal weighting, and each comparison form can be similar to the novel verb in its own way (see *Materials*). In contrast, alternative “structured” measures of similarity (see *Materials*) attend only to phonological properties that are shared uniformly among comparison verbs (e.g., the verb *cling* [klɪŋ] shares its final two segments with *string, sting,* and *fling*, etc.) and thus have the advantage of restricting assessment of similarity to phonological properties that are most relevant to a verb's past‐tense form (e.g., the past‐tense of *cling* is *clung* because it shares a final segment with *string, sting,* and *fling*). With this in mind, one can argue that measures of variegated similarity are penalized for calculating similarity based on any mismatched phonological property across the entire word (i.e., *cling* does not share an initial segment with *string*,* sting,* and *fling*) and can be misled by irrelevant segments of a word. Thus, it is important to investigate whether the effects observed using the variegated measure of similarity in *Analysis 1* are corroborated when using a measure of structured similarity. Table 3 shows the output of a mixed‐effects model that used an identical setup as the mixed‐effects model described above, except that a measure of “structured” similarity was used (obtained from the output of Albright and Hayes's (2003) MGL model; see *Materials*).

**Table 3:** Mixed‐effects models fitted to production of regular forms (“structured” similarity measures obtained from MGL)

	Beta (B)	*SE*	z	2.5%	97.5%	Pr(>|z|)
(Intercept)	−37.61	12.11	−3.11	−61.34	−13.88	0.002
Age	−0.81	1.68	−0.48	−4.10	2.49	0.632
Similarity‐to‐irregulars	−0.11	1.35	−0.08	−2.75	2.54	0.937
Similarity‐to‐regulars	**44.87**	**12.75**	**3.52**	**19.88**	**69.86**	**0.000**
Age*similarity‐to‐irregulars	−0.07	0.20	−0.36	−0.46	0.31	0.716
Age*similarity‐to‐regulars	1.04	1.82	0.57	−2.52	4.60	0.568

Boldface type indicates significant effect.

Consistent with *Analysis 1*, the mixed‐effects model did not show a significant effect of *similarity‐to‐irregulars* but showed the crucial main effect of *similarity‐to‐regulars* in the predicted direction. However, *Age* did not interact with the effect of *similarity‐to‐regulars* **,** nor did it interact with *similarity‐to‐irregulars*. Given that z‐scores (which can be taken as approximate effect sizes) for these interactions were so small (−0.36 and 0.57 for age*similarity‐to‐irregulars and age*similarity‐to‐regulars, respectively), *Analysis 2* does not corroborate the developmental effect observed in *Analysis 1*. On the contrary, *Analysis 2* indicates no developmental change in the effect of *similarity‐to‐regulars*.

In sum, the crucial effect of *similarity‐to‐regulars* was observed regardless of the measure of similarity (variegated [*Analysis 1*] or structured [*Analysis 2*]). One must be cautious about the possibility of any developmental effect: Although the measure of variegated similarity indicated that the effect of similarity‐to‐regulars may decrease with development, the effect did not differ across age groups when a measure of structured similarity was used. Whether one favors a measure of variegated or structured similarity, the data from Analysis 1 and 2 indicate that children produced regular past‐tense forms by analogizing across stored exemplars of existing regular verbs.

## 4 Discussion

Recent judgment and production studies with novel verbs (e.g., Albright & Hayes, 2003; Ambridge, 2010) have provided some evidence that morphological productivity, specifically for the English past‐tense, can be explained by a single‐route analogical process (e.g., Bybee & Moder, 1983), without the need for a context‐free default rule (e.g., Pinker, 1999; Prasada & Pinker, 1993). However, evidence for the crucial effect of similarity‐to‐regulars has been observed only in children aged 9–10 (Ambridge, 2010) and adults (Albright & Hayes, 2003; Ambridge, 2010), with evidence for the former limited to judgment data. This study used a production task (which is presumably less demanding, as well as enjoying greater ecological validity) to investigate whether evidence for the single‐route model extends to younger children (age 3–4; 5–6; 6–7; 9–10); a population who are still in the early stages of morphological acquisition and whose data are perhaps most crucial for any theory of morphological productivity to explain. Past‐tense forms were elicited by showing children an animation of an animal performing a novel action described with a novel verb (e.g., *gezz; chake*) and prompting the child to describe what the animal “did yesterday.” Collapsing across age, the likelihood of a novel verb being produced in regular past‐tense form was positively associated with its phonological similarity to existing regular verbs. Investigation of whether this effect interacted with age revealed that the similarity‐to‐regulars effect was at least as large in the youngest age group as it was in the oldest age group, regardless of whether a variegated similarity metric was used (where each analogical form can be similar to the novel verb in its own way) or a structured similarity metric (where analogy is restricted to focus on phonological properties shared among all analogical forms). Thus, the data suggest that even the youngest age groups were in possession of a morphological system that generates regular past‐tense forms by analogizing across stored exemplars of existing regular verbs.

The results extend previous support (e.g., Ambridge, 2010) for the single‐route model (e.g., Bybee & Moder, 1983; Westermann & Ruh, 2012) to a younger population, and they are inconsistent with the dual‐route model (e.g., Prasada & Pinker, 1993). Specifically, the single‐route model assumes that regular past‐tense forms are generated by analogy across stored exemplars of regular verbs (i.e., the same memory‐based associative process that is posited by both models to be responsible for generation of irregular past‐tense forms). In contrast, the dual‐route model holds that regular past‐tense forms are generated by a separate “add –*ed*” rule, yielding the prediction that regular inflection is not influenced by a verb's phonological similarity to existing regular verbs. Recall that Prasada and Pinker (p. 22) took as evidence for the model their finding that “the goodness of the suffixed past‐tense forms does not decline as a function of distance from known suffixed forms.” Crucially then, only the single‐route model can explain this study's finding that children were more likely to produce a verb with regular inflection when the verb was phonologically similar to existing regular verbs. This finding has important developmental implications for the past‐tense debate because it is the first time a novel verb production paradigm (the most stringent test of the models in question) has been used to demonstrate that overregularization errors made by children as young as 3–4 need not be attributed to a rule‐based mechanism (as under the dual‐route model) and, indeed, are better explained in terms of analogy across stored exemplars (as under the single‐route model).

A possible objection to this conclusion is that while the present finding of an effect of similarity‐to‐regulars on regular inflection is inconsistent with the original “strong” version of the dual‐route model (Prasada & Pinker, 1993), it is not necessarily inconsistent with more recent versions that do allow for some regular storage. For example, Pinker and Ullman (2002, p. 458) state that the model “does not posit that they [i.e., regular past‐tense forms –RB] are *never* stored, only that they do not *have* to be,” thus leaving open the possibility for a modified version of the model that stores and analogizes across regular past‐tense forms (e.g., Alegre & Gordon, 1999; Hartshorne & Ullman, 2006; Ullman & Pierpont, 2005).^6^ In this case, regular past‐tense forms of novel verbs could be generated by rules *or* analogy. Note that such a model is difficult to distinguish empirically from the single‐route model, because—in effect—it *is* the single‐route model (i.e., an analogical system that operates on regulars and irregulars alike) with a default rule bolted on (and no clear specification of when this rule should be used instead of analogy).

However, one possible way to distinguish between the single‐route model and the regular‐storage dual‐route model is as follows. Since, under the latter, irregular past‐tense forms are *always* generated by analogy, while regular forms are *at least sometimes* generated by the default rule instead, the regular‐storage dual‐route model would seem to predict that the effect of similarity‐to‐irregulars on irregular production will be larger than the effect of similarity‐to‐regulars on regular production. The results of this study are not consistent with this prediction: the effect of similarity‐to‐irregulars on the likelihood of irregular (vs. regular) production was numerically smaller than the effect of similarity‐to‐regulars on the likelihood of regular (vs. irregular) production, with only the latter reaching statistical significance. A similar pattern is seen in the adult judgment data reported by Albright and Hayes (2003), with partial *r* values of 0.49 and 0.58 for the effects of similarity to irregulars and regulars, respectively. While further research should attempt to further elucidate the relative effect sizes of irregular and regular analogy, it is already clear that the only way to reconcile the dual‐route model with the findings of this study (and those of Albright & Hayes, 2003; and Ambridge, 2010) is to have it adopt the core mechanism of the single‐route model, making the two almost indistinguishable.

Can a rule‐based account of morphological productivity be salvaged? Most promise comes from considering Albright and Hayes's (2003) MGL model in more detail. Thus far, the present paper has discussed MGL only in terms of its output providing a measure of “structured” similarity that has been used to demonstrate that the likelihood of regular inflection increases with phonological similarity to existing regular verbs—a finding that is consistent with predictions of single‐route models. As outlined in the *Materials* section, MGL forms “micro‐rules” that describe each (word‐specific) phonological change in existing past‐tense forms, and it generalizes over phonological contexts in which existing verbs undergo the same micro‐rule. The model outputs graded confidence values for a verb's past‐tense forms based on the reliability of the context‐dependent micro‐rule which produces that form. The confidence values for each micro‐rule are akin to the output of the connectionist network assumed under single‐route models, which attribute production of past‐tense forms to probabilistic connections between the phonological properties of a verb's stem and past‐tense forms that strengthen based on experience. Alternatively, however, one can interpret the confidence values for each micro‐rule as representative of a more traditional generative linguistic system that consists of “multiple stochastic rules” (e.g., Albright & Hayes, 2003). Such a rule‐based approach is a departure from the “default” rule assumed under dual‐route models because multiple stochastic rules are discovered by an inductive learning mechanism and application of each rule is restricted to specific phonological contexts. Since the similarity‐to‐regulars effect can be explained in terms of either multiple stochastic rules or a connectionist processing system, this study does not favor one of these approaches over the other. Rather, the finding supports the assumption shared by both approaches and which is inconsistent with dual‐route models: that a particular phonological change (e.g., ø→əd) becomes more likely (either through strengthened connections or rule‐based confidence values) with the increasing number of phonologically similar existing regular verbs that undergo the same change.

Future research should investigate whether the more traditional “generative” linguistic system of multiple stochastic rules makes any unique predictions that are not captured by a “connectionist” network. One potentially distinguishable claim is that a system that develops “multiple stochastic rules” as opposed to probabilistic connections comes with a (minimal generalization) mechanism that can develop a “truly default” rule. Such a default rule may explain why English speakers can produce “Handel out‐Bached Bach,” even though “Bach” ends in the velar fricative [x] which is not encountered in the learning data. A “connectionist” model may have more difficulty in accounting for such productions given that its equivalent “default” regularization is produced by high type‐frequency of regular forms, which provides the network with information to abstract the regular pattern.^7^

Although this study was chiefly concerned with investigating the effect of similarity‐to‐regulars, it is also important to consider why an effect of similarity‐to‐irregulars was not observed. One explanation is that the production paradigm primed regular forms (e.g., Ramscar, 2002), thus making children more likely to produce these forms across the board (at the expense of a similarity‐to‐irregulars effect).

To sum up, this study has allowed us to take another step closer to understanding the mechanism that underlies morphological productivity. Elicited production data from children aged 3–4, 5–6, 6–7, and 9–10 revealed that the likelihood of a novel verb being produced in regular past‐tense form was positively associated with its phonological similarity to existing regular verbs. These data are inconsistent with the assumption of the dual‐route model of a context‐free rule that “can apply regardless of anything else that might happen to the stem as a result of other rules or memorized associations” (Berent et al., 2002, p. 463). Rather, it would seem that any account of the developing morphological system must incorporate the assumption that language learners store, and compute phonological analyses across, regular past‐tense forms.

## Notes

1 The present article often refers to this process as an “add ‐*ed* rule” for simplicity, but it is acknowledged that some theorists prefer to describe the process as a concatenation of a symbol for verb‐stem (“V”) and a symbol for the past‐tense morpheme (suffix –*ed*) (e.g., Pinker & Ullman, 2002).

2 Albright and Hayes demonstrated that adults' phonological ratings of stem‐forms were poorly correlated with ratings of past‐tense forms (*r = *0.006), thus indicating that their design successfully minimized any possibility that stimuli had violated rules of English phonology. Thus, this study avoids confounds encountered by a previous study (Prasada & Pinker, 1993) in which a verb like “*ploamphed*” was judged less favorably than “*plipped*”—not because of the former's greater phonological distance from existing regulars, but because it was less well‐formed [i.e., *ploamph/plip*]).

3 Irregular forms of novel verbs were classified based on the irregular form(s) that received the most favorable score in Albright and Hayes's (2003) study. As a consequence of this selection criterion, the irregular form of five verbs was homophonous with the stem or “root” form (e.g., *glit/glit*) on analogy with no‐change forms (e.g., *hit/hit*). Thus, for these five verbs, such forms, when produced, were coded as “irregular.” In the case of some verbs, the model gave a favorable judgment to more than one irregular form of the verb; for such cases, only the form that was most frequently produced (by participants in this study) was coded as “irregular” and the form that was produced less frequently was excluded (note this strategy led to the exclusion of just three trials). See Appendix 1 for a full list of regular and irregular forms.

4 This underlines the point that Figs. 1 and 2, which plot *mean* production scores, should be taken as only a rough indication of the relationship between variables. Since LMEMs predict individual trials rather than averaging over items or participants, a more meaningful interpretation of the relationship between variables is to look at the β values. A positive β value indicates a positive correlation between the predictor and the likelihood of a verb being produced in the relevant form; a negative β value indicates a negative correlation between the predictor and the likelihood of a verb being produced in the relevant form.

5 The present paper does not report analyses for each separate age group because there was not enough power for the effect of *similarity‐to‐regulars* to reach significance for any age group individually (all individual analyses yielded *p* > .1). On the advice of a reviewer, we also analyzed two groups separately (3–6; 6–10) but neither analyses yielded significant effects (*p* > .05).

6 A reviewer noted that a recent paper by an advocate of the regular‐storage model (Ullman, 2013, p. 141) does assume that frequency effects of regular verbs are highly restricted in normal participants: “unlike typically developing control subjects, individuals with SLI show consistent ‘frequency effects’ on regularly inflected past‐tense and plural forms”—and that this “strong” view of the dual‐route model is still often (tacitly) assumed.

7 One possible solution for connectionist models is to posit that a learner can produce *out‐Bached* on the basis of abstracting across instances of other fricatives (i.e., other than non‐velar fricatives; e.g., *hiss, wish*) or by including a semantic component in the analogical process

## Appendix 1

### 1.1

**Table A1:** Core set of 40 novel verbs adopted from Albright and Hayes (2003)^8^

		Regular	Irregular	GCM		MGL		
				(similarity to…)		(similarity to…)		
No.	Verb.	Past	Past	Reg.	Irreg.	Reg.	Irreg.	Verb Set
1	bize	bized	boze	0.530	0.023	0.988	0.121	A
2	blafe	blafed	bleft	0.505	0.001	0.998	0.000	A
3	blig	bligged	blug	0.433	0.067	0.961	0.880	B
4	bredge	bredged	broge	0.369	0.192	0.995	0.000	B
5	chake	chaked	chook	0.403	0.030	0.900	0.835	B
6	chool	chooled	chole	0.576	0.095	0.992	0.000	A
7	dape	daped	dapt	0.532	0.050	0.993	0.000	B
8	dize	dized	doze	0.308	0.000	0.988	0.554	A
9	drice	driced	droce	0.398	0.028	0.998	0.848	A
10	drit	dritted	drit	0.619	0.065	0.944	0.477	A
11	fleep	fleeped	flept	0.653	0.047	0.963	0.848	B
12	flidge	flidged	fludge	0.522	0.000	0.995	0.200	B
13	fro	froed	frew	0.462	0.040	0.943	0.724	B
14	gare	gared	gore	0.686	0.025	0.985	0.257	A
15	gezz	gezzed	gozz	0.657	0.022	0.988	0.000	B
16	gleed	gleeded	gled	0.536	0.012	0.872	0.000	A
17	glip	glipped	glup	0.434	0.000	0.988	0.059	B
18	glit	glitted	glit	0.306	0.017	0.944	0.669	A
19	gude	guded	gude	0.559	0.005	0.989	0.014	A
20	nace	naced	noce	0.464	0.000	0.998	0.046	B
21	nold	nolded	nold	0.228	0.107	0.900	0.014	A
22	nung	nunged	nang	0.546	0.005	0.925	0.000	B
23	pank	panked	punk	0.557	0.030	0.958	0.000	A
24	plim	plimmed	plum	0.374	0.057	0.872	0.348	B
25	preak	preaked	proke	0.507	0.010	0.941	0.033	A
26	queed	queeded	qued	0.507	0.014	0.966	0.411	A
27	rask	rasked	rusk	0.293	0.029	0.982	0.000	B
28	rife	rifed	rofe	0.555	0.040	0.998	0.473	B
29	shilk	shilked	shalk	0.603	0.001	0.982	0.000	B
30	skride	skrided	skrode	0.340	0.017	0.887	0.731	A
31	spack	spacked	spuck	0.448	0.043	0.984	0.000	A
32	spling	splinged	splung	0.437	0.005	0.925	0.880	B
33	stin	stinned	stun	0.417	0.053	0.972	0.280	A
34	stip	stipped	stup	0.352	0.031	0.988	0.149	A
35	stire	stired	store	0.443	0.004	0.985	0.000	A
36	tark	tarked	tork	0.284	0.061	0.982	0.000	B
37	teep	teeped	tept	0.427	0.008	0.963	0.559	B
38	tesh	teshed	tosh	0.585	0.011	0.998	0.000	B
39	trisk	trisked	trusk	0.315	0.034	0.963	0.559	A
40	wiss	wissed	wus	0.470	0.017	0.998	0.000	B
